# Efficacy Evaluation of a VR-2332-Based Modified Live Vaccine Against NADC30-like PRRSV in China

**DOI:** 10.3390/vaccines13050538

**Published:** 2025-05-19

**Authors:** Lixin Li, Xiaxia Tong, Jianhong Shu, Huapeng Feng, Yanping Quan, Yulong He

**Affiliations:** 1 College of Life Sciences and Medicine, Zhejiang Sci-Tech University, Hangzhou 310018, China; 202230903181@mails.zstu.edu.cn (L.L.); shujianhong@zstu.edu.cn (J.S.); fenghuapeng@zstu.edu.cn (H.F.); 2 Zhejiang Hom-Sun Biosciences Co., Ltd., Shaoxing 312000, China; hzhssw@163.com

**Keywords:** porcine reproductive and respiratory syndrome virus, modified live vaccine, efficacy evaluation

## Abstract

**Background:** Porcine reproductive and respiratory syndrome is caused by PRRSV. Modified live vaccines (MLVs) are widely used to control PRRSV infection, but their efficacy against the emerging NADC30-like variant remains unclear. This study aimed to evaluate the efficacy of a VR-2332-based MLV against the NADC30-like PRRSV strain HNjz15. **Methods:** Forty piglets were randomized into a vaccination group (MLV group), negative control group (NC group), and sentinel group. MLV group piglets were immunized with a commercial MLV at 3 weeks of age and challenged with HNjz15 (10^6.6^ TCID_50_/mL) at 21 days post-immunization. Clinical symptoms, viral load, antibody responses, cytokine levels, and lung lesions were monitored for 14 days post-challenge. **Results:** Although fever and respiratory symptoms were more pronounced in the NC group pigs than those of the MLV group (average percent occurrence: 65.2% vs. 52.9%), there was no statistical difference (*p* > 0.05) in the occurrence of respiratory symptoms between the two groups from 5 dpc. Reduced weight gains (by 40–53%) were also observed in the MLV and NC groups compared with the sentinels. The MLV and NC groups exhibited severe lung lesions, while there was no marked difference in viral RNA loads in serum and tissue samples between the MLV and NC groups (*p* > 0.05). The MLV vaccine induced a significant high level of N protein-specific antibodies compared to the NC group. There was also no significant difference in IFN-γ or TNF-α response to the HNjz15 challenge in both groups (*p* > 0.05). **Conclusions:** The VR-2332-based MLV does not provide adequate protection against challenge with the PRRSV-2 NADC30-like strain HNjz15.

## 1. Introduction

Porcine reproductive and respiratory syndrome (PRRS) is the leading swine disease in major pig-producing regions worldwide [1]. It is estimated that PRRS causes approximately USD 664 million in annual economic losses in the United States [2]. A recent Chinese study indicated that the financial loss of PRRS was estimated as CNY 1424 per sow due to a reduced a number of weaned piglets in breeding herds and increased feed cost for fattening herds [3]. PRRSV, a single-stranded positive-sense enveloped RNA virus of the genus *Betaarterivirus* within the family *Arteriviridae*, is the causative agent of this [4]. PRRSV only infects swine and primarily causes high fever and respiratory symptoms in piglets, as well as reproductive failure and fetal death in pregnant sows. Pathological examinations reveal pulmonary edema and hemorrhage with enlarged mediastinal lymph nodes and foamy exudates in the trachea and bronchi, which is indicative of interstitial pneumonia and pulmonary edema [5]. PRRSV infection triggers distinct immunopathological alterations in hosts. A hallmark of this infection is the suppression of critical antiviral mediators, including type I interferons, TNF-α, and IL-1β. Concurrently, natural killer cells (NK cells) exhibit compromised cytotoxic activity, diminishing their capacity to eliminate infected cells. Antibody responses display temporal disparities: non-neutralizing antibodies emerge rapidly in the early stage, whereas neutralizing antibodies develop significantly later. These factors collectively lead to persistent viral infection and the long-term maintenance of viremia [6,7].

PRRS was first reported in the United States in 1987 [8] and has been widespread in North America, Europe, and Asia since the late 1980s [9]. PRRSV-1 is predominant in Europe, whereas PRRSV-2 is most common in North America and Asia [10]. PRRSV-2 is classified into nine distinct lineages based on sequence analysis of the ORF5 gene [11], and the nine lineages can be further divided into sublineages because of the high degree of genetic diversity [11].

PRRSV-2 strains circulating in China are phylogenetically classified into four major lineages [12,13]: lineage 1 (such as NADC30-like and NADC34-like strains), lineage 3 (such as QYYZ and FJFS strains), lineage 5 (such as classical BJ-4 strain), and lineage 8 (such as the highly pathogenic JXA1 and HuN4-F112 strains) [14,15,16]. PRRSV-2 in China can be roughly divided into three epidemiological stages: 1995-2006, with a predominance of lineage 5 strains; 2006–2012, with the emergence of highly pathogenic lineage 8 PRRSV (HP-PRRSV) strains; and 2013 to the present, with a dominance of lineage 1 strains, particularly in central and northern regions [17].

Given the genetic diversity and dynamic evolution of PRRSV, it is increasingly appealing for vaccines to provide broad cross-protection [18,19]. Vaccination is always the primary option for controlling deadly diseases. Currently, the available PRRS vaccines include inactivated vaccines and modified live vaccines (MLVs). Inactivated PRRS vaccines are highly safe but have poor efficacy [20]. The PRRS MLV, introduced in the United States in 1994 [21], is one of the most widely used PRRS vaccines globally and can reduce virus transmission and induce protective immunity. However, it is only effective against homologous strains and poses a risk of recombination with epidemic viruses [22]. Multiple commercial PRRS MLVs against both PRRSV-1 and PRRSV-2 have gained regulatory approval globally. The PRRSV-1-specific vaccine Porcilis^®^ PRRS (MSD Animal Health, Madison, NJ, USA) holds market authorization in Western Europe. The PRRSV-2-targeted Ingelvac^®^ PRRS MLV (Boehringer-Ingelheim, Rhineland-Palatinate, Germany) is licensed in America, Canada, Mexico, China, etc. In China, the PRRS vaccines that are currently in use mainly include lineage 8 live vaccines (e.g., JXA1-P80), lineage 5 live vaccines (e.g., RespPRRS MLV, Ingelvac^®^ PRRS MLV), and lineage 8 inactivated vaccines (e.g., CH-1a) [23,24]. As one of the most widely used vaccines in China, Ingelvac^®^ PRRS MLV, derived from the VR-2332 strain (GenBank no. AF066183.4, lineage 5), has shown noticeable efficacy in reducing the duration of viremia. Despite their widespread application, PRRSV MLVs exhibit safety limitations. Research indicated prolonged viral shedding and sustained replication of the vaccine strain in immunized swine, resulting in persistent viremia and possible horizontal spread to non-vaccinated cohorts [25].

PRRSV-2 strains, especially those of lineage 1, have multiple sublineages that emerge and dominate over time. There are differences in key amino acid sites among these subtypes, which may affect the viral ability to evade the host immune system, leading to the continuous evolution and emergence of new variants. This highlights the complexity of PRRSV control and the importance of immunization with proper vaccines. We hypothesized that antigenic divergence between the VR-2332-based vaccine strain and the NADC30-like strain would compromise the protective efficacy. Considering the widespread prevalence of NADC30-like lineage 1 strains in China, our study was designed to evaluate the immunoprotective efficacy of one commercial VR-2332-based MLV in response to a challenge with a heterologous NADC30-like strain.

## 2. Methods

### 2.1. Viruses, Cells, and Vaccine

The virus utilized for the challenge was HNjz15, an NADC30-like lineage 1 strain of PRRSV (GenBank No. KT945017) [26]. Porcine alveolar macrophages (PAMs) were used for virus growth and titration [27]. The HNjz15 strain has low pathogenicity [27] and was kindly provided by Prof. Kegong Tian (Henan Agricultural University, Zhengzhou, China). Commercial Ingelvac^®^ PRRS MLV (Boehringer-Ingelheim, Rhineland-Palatinate, Germany) was used for the animal trials.

### 2.2. Animals and Experimental Design

All 40 trial piglets that were negative for PRRSV, *Mycoplasma hyopneumoniae*, swine influenza, and PCV2, were purchased from Anji Anxing Husbandry Co., Ltd. (Huzhou, China). The piglets were taken from 6 sows of different parities and transported at 2.5 weeks of age (WOA) to the laboratory animal facility of Zhejiang Novo Biotech Co., Ltd. (China). All procedures and care in this experiment complied with ARRIVE 2.0 [28]. Approval was obtained from Zhejiang Sci-Tech University’s IACUC (No. 20220623-01), with all animals being humanely euthanized via a Zoletil™50 overdose (10 mg/kg). No animals were excluded from analysis, and no adverse events occurred during the study.

All the piglets were weighed, ear-tagged, and randomized into three groups based on litter, weight, and sex: a vaccination group (MLV group, n = 15), negative control group (NC group, unvaccinated but challenged group, n = 15), and sentinel group (without vaccination and challenge, n = 10). At 3 WOA, the MLV group piglets were immunized intramuscularly (IM) with 2 mL of the vaccine, with the time set as day 0 post-immunization (dpi). The NC and sentinel groups were given 2 mL of sterile phosphate-buffered saline (PBS) intramuscularly. At 21 dpi, piglets in both the MLV and NC groups were challenged via two routes: 2 mL of the HNjz15 virus suspension (10^6.6^ TCID_50_/mL), administered as 1 mL per nostril intranasally (IN), followed by 1 mL IM injection (Table 1).

### 2.3. Clinical and Macroscopic Observations

Local and systemic signs were monitored daily for the first 7 days after inoculation. The rectal temperatures of all piglets were measured and recorded for 8 days after immunization (0–7 dpi) and for 14 days after the challenge (i.e., 21–34 dpi or 1–14 days post-challenge [dpc]). Pigs were weighed at 0, 21, 28, and 35 dpi to calculate the average daily weight gain (ADWG). Following the viral challenge, pigs were monitored daily in terms of their physical conditions and clinical symptoms, they were scored based on behavior, and their respiratory symptoms were recorded daily [28,29,30]. Macroscopic lung lesion scores were assessed as previously described [31]. Each lung lobe was assigned scores from 5 to 15 based on the proportion of affected lung volume. The cumulative score across all lobes provided a comprehensive assessment, with the total values spanning 0 to 100 points [32].

### 2.4. Sample Collection

Serum samples were collected at 0, 7, 14, 21, 28, and 35 dpi for detection of viremia and antibody responses. Blood samples were collected in sterile tubes and centrifuged at 2000× *g* for 10 min for the collection of sera. The serum samples were stored at −80 °C in 1 mL aliquots. At 35 dpi, all animals were humanely euthanized with Zoletil™50 (Virbac, Carros Cedex, France) for subsequent necropsies to score pulmonary lesions. The carcasses were then transferred to DADI WEIKANG Company (Zhejiang, China) for environmentally compliant incineration in accordance with biosafety regulations. During the necropsy, lung and thoracic lymph node specimens were aseptically collected. A 10% (*w*/*v*) tissue homogenate was prepared in PBS and centrifuged (4000× *g*, 10 min), and supernatants were stored at −80 °C for subsequent viral RNA extraction. Parallel tissue sections were fixed in 10% neutral buffered formalin for hematoxylin–eosin (HE) staining and immunohistochemistry (IHC).

### 2.5. Viral Load

RNA was extracted from the serum samples and homogenates of the tissues using a nucleic acid extraction system (HERO32, Luoyang Ascend Biotechnology, Luoyang, Henan, China). Viral RNA copies were quantified using the TaqMan^®^ probe-based PRRSV-specific nucleic acid detection kit (Daan Gene, Guangzhou, China, lot: 20210823) on the ABI 7500 real-time PCR system (Applied Biosystems, Foster City, CA, USA). The primers were as follows: F: 5′-CCCTCATCAACCAATGTCACG-3′; and R: 5′-GACAGACTCCGCTTTTCGAGAC-3′. The TaqMan probe was synthesized as FAM-ACCCAAAGGTCTCCGCCGGAATTC-BHQ. The manufacturer-recommended protocol included reverse transcription at 50 °C for 15 min, denaturation at 95 °C for 15 min, and 40 amplification cycles of 94 °C for 15 s and 55 °C for 45 s. Absolute quantification was achieved using a serial 10-fold dilution series (10^3^ to 10^8^ copies/μL) of PRRSV RNA standards, provided by the kit manufacturer. These standards were derived from full-length PRRSV genomic RNA, calibrated by ultraviolet spectrophotometry and validated against established reference materials. RNA copies were transformed to log_10_ values for statistical analysis. 

### 2.6. Analysis of Serum Antibody Response and Cytokines

The IDEXX PRRS X3 ELISA test kit (IDEXX Laboratories, Inc., Westbrook, ME, USA, lot: AA851) was employed to assess antibody responses against PRRSV N protein following the manufacturer’s protocol. Seropositivity was defined as a sample/positive (S/P) ratio ≥ 0.4. The sera were obtained at 0, 7, 14, 21, 28, and 35 dpi. Subsequently, the concentrations of interferon gamma (IFN-γ) and tumor necrosis factor alpha (TNF-α) in the serum samples were quantitatively determined using Porcine IFN-gamma ELISA Kit (Invitrogen™, Carlsbad, CA, USA, lot: 285479-002) or Porcine TNF-alpha Quantikine ELISA Kit (R&D Systems, Inc., Minneapolis, MN, USA, lot: P289002). All experimental procedures adhered to the manufacturer’s protocols.

### 2.7. Histopathology and Immunohistochemistry Staining

For the microscopic lung lesion score, the samples were embedded in paraffin, and the sections were stained using the HE method as previously described [33] and evaluated under a light microscope. A histopathological evaluation of lung tissues was performed by blinded investigators using a semi-quantitative scoring system (0–5 scale) [32]. The scores reflect progressive pulmonary interstitial inflammation, where 0 denotes normal alveolar architecture and 5 indicated diffuse inflammatory infiltration with septal thickening. The viral antigen distribution was visualized through immunohistochemical staining (IHC) with a specific anti-PRRSV N protein antibody [33].

### 2.8. Statistical Analysis

Temperature, cytokine, and antibody data, viral load over time were analyzed using one-way repeated measures ANOVA (one-way RM ANOVA). The average daily weight gain was tested by one-way ANOVA. Discrete variables, including lung lesion score, and the microscopic lung lesion score were evaluated via the nonparametric Kruskal–Wallis test [34]. The percentage of pigs showing scored respiratory symptoms was analyzed by the Chi-square test. Statistical analyses were conducted using IBM SPSS Statistics v25 (IBM Corp., Armonk, NY, USA) and GraphPad Prism v9.0 (GraphPad Software, San Diego, CA, USA).

## 3. Results

### 3.1. Clinical Symptoms and Pig Growth Performance

**Figure 1:**
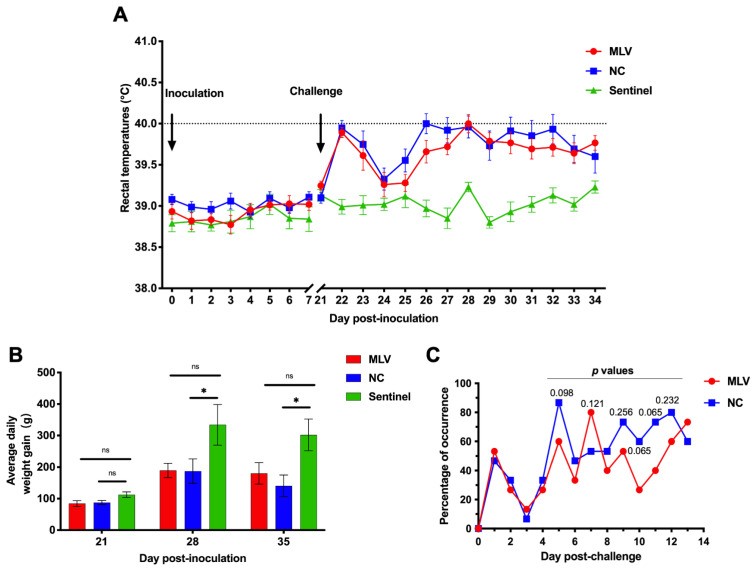
Rectal temperature profiles, average daily gain, and occurrence of respiratory symptoms of the piglets in the MLV, NC, and sentinel groups. (**A**) Rectal temperatures: ≥ 40.0 °C were considered to indicate fever. Arrows indicate the time of MLV inoculation or challenge with strain HNjz15. (**B**) Average daily weight gain at 21, 28, and 35 dpi, equivalent to day 0, 7, and 14 post-challenge with the HNjz15 strain. ns, *p* > 0.05; *, *p* < 0.05 (one-way ANOVA). (**C**) Percentage of occurrence of respiratory symptoms, coughing, tachypnea, or dyspnea (with scores of 2–3) in 15 piglets of the MLV or NC group after the challenge (*p* > 0.05, Chi-square test).

During the initial seven days post-inoculation, there was no significant difference in rectal temperatures, which were relatively stable and similar, among all the piglets in the three groups (Figure 1A), indicating that MLV immunization itself did not elicit a noticeable fever response. After the challenge with the NADC30-like strain HNjz15, the rectal temperatures in the MLV and NC groups increased significantly compared with the sentinel group. Specifically, all piglets in both the MLV and NC groups exhibited rectal temperatures exceeding 40.0 °C for at least two days (Figure 1A). Temperatures were generally higher in the NC group than in the MLV group, but without statistical difference (*p* > 0.05). The highest mean rectal temperatures occurred at 26 dpi or 5 dpc (40.0 ± 0.47 °C) in the NC group and 28 dpi or 7 dpc (40.0 ± 0.43 °C) in the MLV group. The ADWGs in the MLV and NC groups were lower than that of the sentinel group, with an about 44% reduction in both groups compared with the sentinels (334.3 g) at 7 dpc, and 40% (MLV group) or 53% (NC group) reductions compared with the sentinels (302.1 g) at 14 dpc, where there was statistical significance between the NC group and sentinel group (*p* < 0.05, Figure 1B). There was no difference in the ADWG between the MLV and NC groups at any time point.

In general, the challenge virus HNjz15 did not elicit severe clinical symptoms. The symptoms were mild and included fever ≥40.0 °C, anorexia, coughing, and tachypnea or dyspnea, scored at levels 2 to 3. Not all pigs in the MLV and NC groups exhibited respiratory symptoms. The number of pigs showing such symptoms ranged from 26.7% to 86.7% in the MLV and NC group at 5–14 dpc. While the average occurrence was lower in the MLV group (52.9%) than the NC group (65.2%) during the period of 5–14 dpc, there were no statistical differences (*p* > 0.05 at indicated time points) between the two groups (Figure 1C). Throughout the study, piglets in the sentinel group showed no clinical signs. The above findings suggest that the vaccine failed to offer protection against the viral challenge at the clinical level.

### 3.2. Pathological and Histopathological Findings

**Figure 2:**
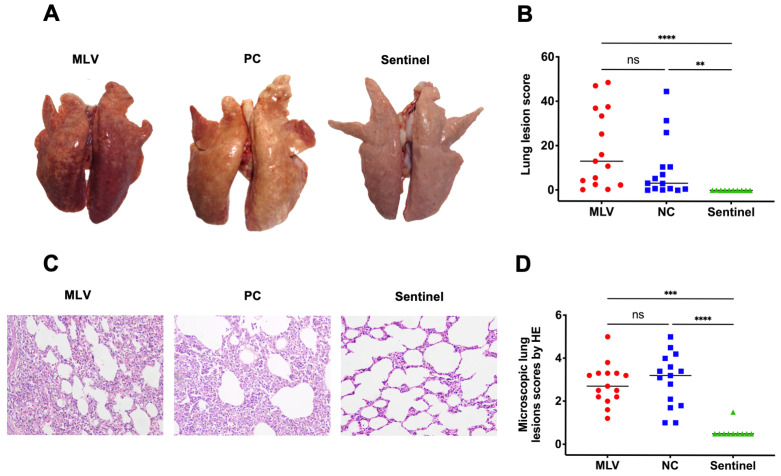
Macroscopic and microscopic lesions from piglets in the MLV, NC, and sentinel groups. (**A**) Representative lung samples. (**B**) Scores of the macroscopic lung damage. (**C**) Representative microscopic images showing the lung lesions. (**D**) Scores of the microscopic lung lesions. ns, *p* > 0.05; **, *p* < 0.01; ***, *p* < 0.001; ****, *p* < 0.0001 (Kruskal–Wallis test). N = 15 piglets for the MLV or NC group, and N = 10 for the sentinel group.

The lungs of the piglets that were challenged with the NADC30-like strain HNjz15 presented typical lesions, including mottled-tan-red consolidation, although the extent of these lesions varied among individuals (Figure 2A). Lymph nodes were generally swollen and tan in appearance. The piglets in the MLV and NC groups showed significant lung damage, with a high percentage exhibiting severe lung lesions that were significantly different from those in the sentinel group (Figure 2A,B), and there was no apparent difference in lung lesion scores between the MLV and NC groups (*p* > 0.05, Figure 2B). Enlarged lymph nodes and tonsils were also observed. Several pigs in the MLV and NC groups exhibited interstitial pneumonia, characterized by thickened alveolar walls and infiltration of inflammatory cells that are typical of the histopathological features of PRRSV infection (Figure 2C). No significant differences in the severity of histopathological lung lesions (with scores on a scale of six levels) were observed between the MLV and NC groups, while both groups exhibited significantly more severe lesions compared to the sentinel group (Figure 2D).

### 3.3. Antibody Responses

**Figure 3:**
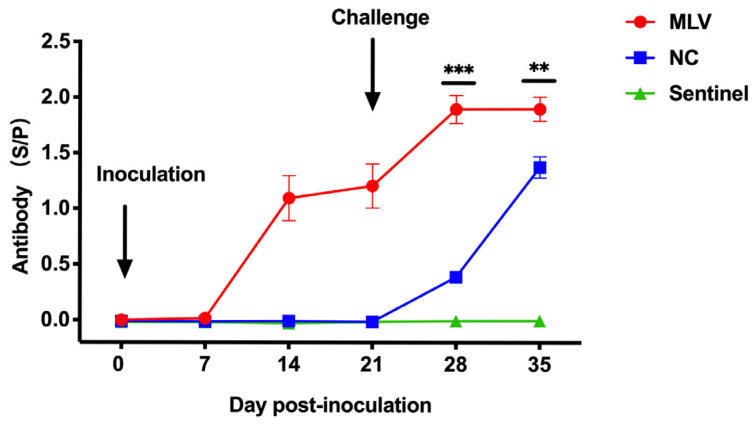
Antibody levels shown as S/P ratio in sera of piglets in response to MLV immunization and challenge with the HNjz15 strain. **, *p* < 0.01; ***, *p* < 0.001 (one-way RM ANOVA). N = 15 piglets for the MLV or NC group, and N = 10 for the sentinel group.

The antibody responses against PRRSV N protein were detected by ELISA in the sera collected at weekly intervals up to day 35 after vaccination. There were no antibodies detected in any group at 7 dpi, and the sentinel group remained antibody-negative throughout the study period (Figure 3). At 14 and 21 dpi prior to the challenge, all piglets (15/15) in the MLV group were seropositive for the N protein, with antibody levels that were significantly higher than those in the NC group from 14 to 35 dpi (*p* < 0.01 or <0.001). The HNjz15 virus challenge further increased N protein antibody levels in the MLV group at 7 and 14 dpc and also triggered an antibody response in the NC group.

### 3.4. Cytokines

**Figure 4:**
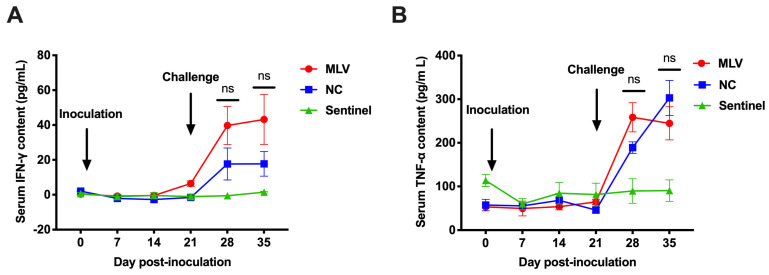
Concentrations of IFN-γ (**A**) and TNF-α (**B**) in sera of piglets in response to MLV immunization and challenge with HNjz15 strain. ns, *p* > 0.05 (one-way RM ANOVA). N = 15 piglets for the MLV or NC group, and N = 10 for the sentinel group.

A serum cytokine analysis was performed to evaluate the systemic Th1/Th2 immune balance and inflammatory status after the challenge. The concentrations of IFN-γ (a Th1-associated cytokine) and TNF-α (a pro-inflammatory mediator) were quantified longitudinally in all groups (Figure 4). There were no inter-group differences in the pre-challenge IFN-γ and TNF-α level (*p* > 0.05). Following the HNjz15 virus challenge, the IFN-γ concentrations in the MLV and NC groups increased moderately, peaking at 43.21 pg/mL (MLV group) and 17.63 pg/mL (NC group) by 14 dpc (35 dpi), but without statistical difference between the two groups (*p* > 0.05). The TNF-α response in the MLV pigs displayed an early peak of 258.46 pg/mL at 7 dpc, with a slight decrease at 14 dpc, whereas the NC animals showed progressive elevation from 189.06 pg/mL to 303.12 pg/mL. There were no statistical differences between the MLV and NC groups (Figure 4, *p* > 0.05). The concentrations of both IFN-γ and TNF-α in the sentinel group remained low throughout the study period.

### 3.5. Viremia and Tissue Viral Load

**Figure 5:**
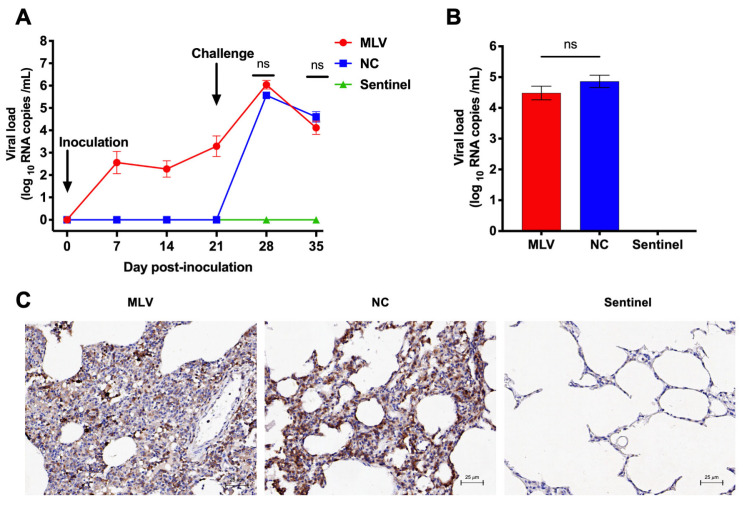
PRRSV RNA copies in sera and mixed tissues of lungs and lymph nodes of the piglets in response to MLV vaccination and challenge with the HNjz15 strain. (**A**) Time-course of the viral RNA copies in sera. (**B**) Viral RNA copies in the mixed lung and lymph node samples at necropsy (35 dpi or 14 dpc). ns, *p* > 0.05 (one-way RM ANOVA). (**C**) Detection of the viral antigen in the lung samples by IHC. N = 15 piglets for the MLV or NC group, and N = 10 for sentinel group.

Viral RNA was readily detectable in the majority of the vaccinated piglets from the MLV group, with 14/15 (93.3%), 15/15 (100%), and 15/15 (100%) being positive at 7, 14, and 21 dpi, respectively (Figure 5A). Both the MLV and NC groups exhibited a marked increase in viral RNA loads at 7 dpc. Before the challenge, viral RNA was not detected in the NC pigs. Elevated viral loads in the piglets of the MLV and NC groups were observed at 7 and 14 dpc, with no statistical difference (*p* > 0.05). No viral RNA was detected in the sera of the sentinel group throughout the study (Figure 5A). Consistent with their viremia profiles, high viral RNA levels were detected in mixed samples of lungs and lymph nodes from all challenged piglets, with no statistical difference between the MLV and NC groups (*p* > 0.05, Figure 5B). No viral RNA was detected in the tissues of the sentinel pigs. The immunohistochemistry further confirmed virus-positive cells, detected in the lung samples of all five pigs in the MLV group or the NC group, whereas the sentinel group’s lungs remained negative (Figure 5C).

## 4. Discussion

The evolving nature of PRRSV leads to the emergence of novel variants such as NADC30-like or NADC34-like strains [23]. According to a report by Yuan et al. (2025), nearly 50% of thesequences analyzed belonged to sublineage 1.8, and about 20% belonged to sublineage 1.5 (NADC34-like), in samples collected from several northern Chinese provinces [35]. The fact that PRRSV NADC30-like strains are becoming increasingly prevalent in China further highlights the need to evaluate the performance of current vaccines [17,36].

The efficacy of the VR-2332 MLV in significantly reducing the prevalence of persistently infected pigs with homologous strains has been well documented [37,38]. There were studies showing that the MLV provides cross-protection against heterologous strains of the same type of PRRSV-1 or PRRSV-2 [1,39,40]. This might be one of the reasons why MLVs have been widely used, regardless of the genetic divergence of the virus strains over time. However, the performance of the VR-2332-based MLV against heterologous PRRSV strains, such as those of the NADC30-like lineage 1, remains a topic of ongoing research and debate [41]. While the vaccine has been reported to have some efficacy against some heterologous strains [30,42], its cross-protective efficacy remains uncertain [28,37,41,43].

In our study, the efficacy of the VR-2332 MLV was assessed through a multidimensional evaluation framework in piglets challenged with the HNjz15 strain. The parameters included clinical assessments and scores of macroscopic pathological and histopathological lesions. A phylogenetic analysis was used to classify HNjz15 as NADC30-like, having 95.6% nucleotide identity with the NADC30 strain and 89.6% identity with the HP-PRRSV JXA1 strains [33]. Our study indicated that although vaccinated pigs exhibited less severe clinical signs than non-vaccinated control animals in response to the challenge with the HNjz15 strain, the protection was incomplete. Vaccinated pigs still demonstrated elevated body temperatures, reduced weight gain, and mild respiratory dyspnea, and the viral loads in the MLV and NC groups were similar in the blood at 7 and 14 dpc, as well as in tissues at 14 dpc. These findings suggest that the VR-2332-based MLV failed to provide discernible protection against challenge with the NADC30-like strain HNjz15. Our results are generally consistent with the findings for lineage 1 MLVs [41] and lineage 5 MLVs [43,44] against other NADC30-like strains, but different from the research reported for lineage 5 MLVs [30] and lineage 8 MLVs [42].

The failure of cross-protection against heterologous strains might be attributed to lower genomic similarity or antigenic variations between the MLV strain and the NADC30-like strains that are used for the challenge [37]. Li et al. reported that a commercial MLV differed significantly in its efficacy against two strains: it was more effective on the HP-PRRSV of higher genomic similarity (95.3% nt) than on the NDAC30-like strain (85.9% nt), including the epitopes A, B, and C of ORF5 that were present in the former but absent in the latter [41]. Serum neutralization assays demonstrated that sera from MLV-vaccinated animals exhibited a slight promoting effect on homologous HP-PRRSV replication. In contrast, significant enhancement of viral replication was observed for heterologous NADC30-like PRRSV lineage 1 strains in vitro. These findings indicated that antibody-dependent enhancement (ADE) might be one of the factors contributing to the diminished protective efficacy of MLVs [28], as documented in vivo in the 1990s [45]. Notably, antibodies targeting the N protein may also contribute to ADE [46,47,48], which might contribute to the failure of protection observed in our study.

Viremia in pigs who were immunized with the MLV persisted for at least 3 weeks or even longer (up to 4 weeks) [37]. This can lead to virus shedding and transmission to pigs or those who are already infected with field strains, creating a condition that may promote virus recombination and the generation of novel strains. The incomplete cross-protective immunity conferred by MLVs against NADC30-like PRRSV variants may explain their widespread transmission in Chinese swine herds [17,26,42]. Because of different recombination among different PRRSV strains in the fields, the NADC30-like strains may also differ in pathogenicity [49].

Cytokines are one of the main indicators for measuring cellular immunity, particularly the interferons (IFNs) that play a crucial role in the innate immune response to viral infections [50]. IFN-γ is essential for macrophage activation and antiviral defense, while TNF-α from activated macrophages regulates the functions of infected cells, including cell proliferation, survival, differentiation, and apoptosis [50,51]. Elevated IFN-γ and TNF-α levels exhibit correlation with both clinical symptoms and histopathological improvement in PRRSV challenge models, regardless of the viral strain homology [52]. Studies have shown that the early peak of IFN-γ level is closely related to the reduction in PRRSV-2 viremia, while TNF-α exhibits a biphasic distribution, reaching its initial peak at 7 dpi. Although TNF-α is associated with pro-inflammatory responses, its brief increase is consistent with a decrease in TGF-β expression, indicating its role in counteracting immunosuppressive signals [53]. In our study, serum IFN-γ and TNF-α were not induced by MLV immunization. However, the pigs responded to the viral challenge with increased secretion of both cytokines in blood. Although IFN-γ was higher in the MLV group than the NC group, there was no statistically significant difference, suggesting that MLV vaccination could not activate an IFN-γ response in response to the challenge. This is somehow different from a report by Jeong et al., who reported that MLV immunization initiated markedly higher frequencies of IFN-γ-secreting peripheral blood mononuclear cells than the unvaccinated controls using the ELISPOT assay [54]. The lower sensitivity of the ELISA method compared with ELISPOT in IFN-γ assays might account for the difference. Because an ELISPOT analyzer was not available in our laboratory, we chose the ELISA kit to measure serum IFN-γ levels instead, which was used in previous studies in similar contexts [41,55].

PRRSV-specific neutralizing antibody (NA) was not examined in this study for the following reasons: Previous studies have demonstrated that serum antibodies with PRRSV-neutralizing activity typically do not appear at 28 dpi [52,55,56,57,58]. In vitro NA titers measured on non-target cells (e.g., MARC-145) correlate poorly with in vivo neutralization capacity in PAMs [48], questioning their biological relevance. Heterologous challenges often fail to elicit cross-reactive NAs due to PRRSV’s immune evasion strategies [54,56,59] or NA titers that are not correlated with the viral clearance in heterologous challenges [56,60], rendering NA detection non-predictive of protection.

Future research should focus on the antigenic epitopes in major structural proteins, such as GP5 and M, of the NADC30-like strains that induce neutralizing antibodies. Understanding mutations in these critical epitopes is important for designing vaccines that can effectively neutralize emerging strains. Priority should be given to strain-matched vaccine development. Reverse genetics can be used to modify vaccine strains to enhance their cross-protection ability by chimeric neutralizing epitopes. Exploring novel adjuvants and delivery systems could significantly improve the breadth and strength of vaccine immune responses. Developing multivalent vaccines targeting multiple PRRSV strains or genotypes may offer broader protection. Long-term follow-up studies are required to assess the memory immune responses after vaccination and their protection against reinfection with prevalent strains. Longitudinal assessments of immune markers, such as neutralizing antibodies and T-cell responses, will provide valuable data on the durability and effectiveness of immune memory. These combined advancements are critical for improving PRRSV control in the swine industry.

## 5. Conclusions

We demonstrated that the commercial VR-2332-based MLV fails to confer effective protection against the NADC30-like PRRSV strain HNjz15 under experimental conditions. This study, together with the findings of others, underscores the imperative to evaluate the cross-protective efficacy of widely used commercial MLVs against genetically diverse PRRSV strains that are prevalent in China, such as the lineage 1 NADC30-like strains.

## Figures and Tables

**Table 1:** Vaccination and challenge of piglets *.

Group	Number of Piglets	Vaccination	Route and Dose of Inoculation	Challenge	Route and Dose of Challenge
**MLV**	15	MLV	2 mL IM	HNjz15	1 mL IN plus 1 mL IM
**NC**	15	Sterile PBS		HNjz15	
**Sentinel**	10	Sterile PBS		Sterile PBS	

* IM: intramuscularly; IN: intranasally.

## Data Availability

All data generated or analyzed during this study are included in this published article. The datasets used and/or analyzed during the current study are available from the corresponding author on reasonable request.

## Abbreviations

ADWG	average daily weight gain
Dpc	days post-challenge
Dpi	days post-inoculation
IN	intranasally
IM	intramuscularly
IFN-γ	interferon gamma
MLV	modified live vaccine
RT-PCR	real-time polymerase chain reaction
PRRS	porcine reproductive and respiratory syndrome
PRRSV	porcine reproductive and respiratory syndrome virus
TNF-α	tumor necrosis factor-alpha
WOA	weeks of age
